# Lipoprotein (a), an independent cardiovascular risk marker

**DOI:** 10.1186/s40842-016-0024-x

**Published:** 2016-03-31

**Authors:** Ramesh Saeedi, Jiri Frohlich

**Affiliations:** 1 Healthy Heart Program, St. Paul’s Hospital, Rm 180-1081 Burrard Street, Vancouver, B.C V6Z 1Y6 Canada; 2 Pathology and Laboratory Medicine Department, University of British Columbia, St. Paul’s Hospital, Rm 180-1081 Burrard Street, Vancouver, B.C V6Z 1Y6 Canada

**Keywords:** Lipoprotein(a) [Lp(a)], Coronary artery disease (CAD), Niacin, Statins

## Abstract

Epidemiological and genetic studies have identified elevated levels of lipoprotein (a) ((Lp(a)) as a causal and independent risk factor for cardiovascular diseases (CVD). The Lp(a)-induced increased risk of CVD may be mediated by both its proatherogenic and prothrombotic mechanisms. Several guidelines recommend screening of Lp(a) level; however, there are few treatment options for the management of patients with elevated Lp(a). Several new medications for Lp(a) are under development. PCSK9 inhibitors, apolipoprotein (a)-antisense, and apolipoprotein(B-100)-antisense mipomersen have shown promising results. Lp(a) reduction will continue to be an active area of investigation.

## Background

Most clinical trials research investigating the effects of cholesterol lowering medications on the prevention of cardiovascular disease (CVD) have focused on low density lipoprotein cholesterol (LDL-C). Elevated serum level of lipoprotein (a) (Lp(a)), an LDL particle linked to the plasminogen-like glycoprotein, has been an independent risk factor for atherosclerotic CVD, particularly in those with high low-density lipoprotein cholesterol (LDL-C) or non- high-density lipoprotein cholesterol (HDL-C) [1, 2]. Effects of Lp(a) on vasculature are not fully understood. Human and animal studies have shown that Lp(a) can enter intima of arteries [3]. Thus, it may have a role in inflammation of intima, thrombosis, and foam cell formation; all these processes are involved in development of atherosclerosis [4, 5]. It is estimated that around 1.5 billion people have Lp(a) levels greater than 500 mg/L [6]. Lp(a) levels are, to a large extent genetically determined, stable are not significantly influenced by diet, exercise, or other environmental factors [6].

This review article addresses the structure, genetics, and function of Lp(a) as well as indications for screening and therapy.

### Lp(a) structure

The Lp(a) structure is similar to that of LDL, in which a glycoprotein, apolipoprotein(a) [apo(a)], is covalently bound to apolipoprotein B (apoB) by a disulfide bridge, in a 1:1 molar ratio [2]. Cholesterol content of Lp(a) as well as its density are similar to those of LDL particles. Its structure is similar to plasminogen, including a common gene sequence [2]. The apo(a) chain contains five domains or kringles; the fourth kringle has a homology with the fibrin-binding domain of plasminogen which causes Lp(a) interference with fibrinolysis. In addition, Lp(a) promotes foam cell formation and cholesterol deposition in atherosclerotic plaques by binding to macrophages [7].

### Lp(a) genetics and serum concentration

Serum levels of Lp(a) are mostly genetically determined. The polymorphism in apo(a) gene [LPA] results in the heterogeneity in its size and molecular weight [1, 2]. Lp(a) show high ethnic variability. Atherosclerosis Risk in Communities (ARIC) study has shown that median Lp(a) levels are three times higher in African-Americansas compared to the Caucasions [8]. Matthews et al. [9] also reported higher levels of Lp(a) in African-American compared to Caucasians.

Distribution of Lp(a) concentrations is highly skewed particularly toward extremely high levels [2, 7, 10]. Lp(a) levels range between 20 to more than 2000 mg/L with almost 20 % of individuals at the extreme levels [2, 7, 10].

There is a heterogeneity in size of Lp(a), ranging from 187 to 662 KDa, it depends on the number of Kringle of IV type 2 repeats in the Lp(a) gene [11]. There is a strong inverse relationship between the size of Lp(a) and its serum levels with smaller size correlating with higher serum levels [2]. Also smaller Lp(a) is associated with increased risks of cardiovascular disease (CVD). Due to its strong genetic determination, Lp(a) levels are stable and are not significantly influenced by gender, age, or environmental factors [7, 10].

### Lp(a) measurement

Originally Lp(a) was detected by gel electrophoresis as a “sinking pre-beta lipoprotein” band [2]. There are issues regarding Lp(a) measurement and standardization which have complicated Lp(a) level interpretations in the context of CVD risk. There are several diferent methods of reporting Lp(a) leves that may confuse physicians. Some laboratories report Lp(a) mass, while others report it as Lp(a) concentration, or Lp(a) protein. Efforts has been made in standardization on measurement and reporting the Lp(a) levels. Several assays for measurement of Lp(a) are available, such as enzyme-linked immunosorbent assays (ELISAs), non-competitive ELISA, immunonephelometry, immunoturbidometry, and latex immunoassays [11–13]. The European Atherosclerosis Society guidelines [12] recommended use of method which is robust, accurate, economically priced with coefficient of variations (CV) <10 %. Also they have recommended that antibodies which are used in assay kits should be apo(a) isoform-insensitive in which Lp(a) is measured independently of Lp(a) size and number of kringle-IV repeats. Immunoassays, protocols for blood collection, plasma/serum isolation should be standardized for quality control. A secondary reference Lp(a) preparation at an international level approved organizations such as International Federation of Clinical Chemistry and the World Health Organization. Manufactures making assays for measurement of Lp(a) should seek to minimize the effects of apo(a) size on Lp(a) levels and use isoform-insensitive polyclonal antibodies (anti-apo(a) capture and anti-apo(B) [11–15].

Denka-Seiken immunoturbidimetric assay (Atherotech Diagnostics Lab; Berkeley Extended-Range Lp(a) Test) is a high-duality and validated assay that measures Lp(a) mass with CV <3 %.

### Lp(a) and cardiovascular disease (CVD)

Serum levels of Lp(a) are an independent risk factors for CVD. Several studies have indicated that the risk is markedly elevated at the extreme Lp(a) levels [16, 17]. For example, the Copenhagen Heart Study showed that patients with Lp(a) levels above 500 mg/L have 2–3 fold increase in the risk of myocardial infarction. A large Mendelian randomized study as well as the EPIC-Norfolk, and Brunek cohort studies demonstrated that patients with high Lp(a) levels have two-fold increase in CVD [7]. Recently Willeit et al. suggested that Lp(a) measurement, particularly in intermediate risk category of patient (as determined by Framingham risk score) predicts CVD outcomes and improves CVD risk prediction [18].

HIgh levels of Lp(a) are commonly detected in patients with premature coronary heart disease (CHD). Genest have reported elevated Lp(a) levels in 18.6 % of patients with premature CHD with 12.7 % of them having no dyslipidemia [19]. The National Cholesterol Education Program for the Detection, Evaluation and treatment of hypercholesteremia in adult (NCEP-ATP III) states that Lp(a) levels are predictor of CV risks with higher levels associated with greater risk of having an CV events [20]. The Emerging Risk Factors Collaboration have found that each 3.5-fold increase in Lp(a) resulted in a 13 % increase in CVD risk [21]. They also found that this association was continuous and become proportionally more important with higher Lp(a) levels. Moreover, they found that this association still persist even after correction for other lipid parameters.

Size of Lp(a) also modulates CHD risk with the smaller apo(a) isoforms associated more strongly with the risk of CHD compared to larger isoforms [21]. Genome-wide association studies (GWAS), Mendelian randomization, and epidemiologic studies revealed a link between LPA genotype with high Lp(a) levels and adverse CV events [6]. In a systemic review of 40 studies involving 58,000 participants, Erqou et al. have shown that people with smaller apo(a) isoforms have approximately 2-fold higher risk of CHD or ischemic stroke than those with larger isoforms [21]. Interestingly, they found that after adjustment for cholesterol and other CVD risk factors, the association was only slightly attenuated, strengthening the concept that Lp(a) is an independent risk factor for CHD. Likewise, European Prospective Investigation of Cancer cohort have observed that after adjustment for LDL-C, there is an association between Lp(a) levels and CVD [7]. Recently, Khera et al. analysed a subgroup of white participants of the JUPITOR study and showed that in statin-treated patients with very low level of LDL-C, elevated Lp(a) levels represent a significant determinant of residual risk for CVD [22].

LPA gene polymorphism influences Lp(a) levels and the risk of myocardial infarction with some common variants of LPA gene associated with more than 50 % risk of heart diseases [1, 2]. Two gene association studies have suggested a causative relationships between elevated levels of Lp(a) and increased risk of CVD events.

The 10 years Framingham cardiovascular risk score does not include Lp(a) levels. However, including it may improve risk assessment. To support this idea, several cohort of patients (both Caucasian and African-American patients) were used to develop 2013 American College of Cardiology/American Heart Association (ACC/AHA) cardiovascular risk calculator [20]. This model incorporated same parameters as the 2008 Framingham cardiovascular risk score except that it included only hard endpoints (both fatal and not fatal myocardial infarction and stroke). Thus, this calculator may be used in some populations; while it is not accurate in others (Rotterdam) [23, 24].

In addition, elevated levels of Lp(a) are associated with stroke, which is more common in men than women. Elevated Lp(a) in patients with essential hypertension seems to play a role in the development of target-organ damage. In one study, Sechi et al. have shown that regardless of blood pressure, Lp(a) levels were the best predictor of target-organ damage involving arterial wall, heart, and kidney [25].

Lp(a) may also play a role in plaque rupture and coronary thrombosis[23–26]. In patients with acute coronary syndrome, Lp(a) levels are predictive of risk of cardiac death.

### Screening

Recommendations for Lp(a) screening are not standardized among atherosclerosis, lipid, and CV prevention societies. The 2013 ACC/AHA treatment guideline did not examine Lp(a) role and thus did not recommend Lp(a) screening [26]. However, both the National Lipid Association (NLA) and the European Atherosclerosis Society Consensus Panel recommended Lp(a) measurement for patients with familial hypercholesteremia, strong family history of CVD and/or elevated Lp(a), personal history of premature CVD, recurrent CVD despite statin treatment, inadequate response to statins, and ≥ 3 % 10-year risk of fatal CVD according to the European guideline, and ≥ 10 % 10-year risk of fatal or non-fatal CHD according to the US guidelines [12, 27]. The 2012 Canadian Cardiovascular Society recommend considering secondary testing, including Lp(a) measurement, in a moderate risk patients [28]. They emphasize that LDL-C reduction is the primary target in management of dyslipidemia.

### Cardiovascular risk in patients with elevated Lp(a)

There is no definite clinical trial investigating effect of lowering Lp(a) on prevention of CHD; thus, all recommendations for the treatment are speculative. Thus ACC/AHA guidelines does not identify Lp(a) as the primary target for lipid-lowering therapy [26]. The European Atherosclerosis Society Consensus Panel (EASCP) suggested that Lp(a) level below 500 mg/L are desirable [12]. The primary goal of treatment of elevated Lp(a) is to lower LDL-C to the patient’s target LDL-C level based on patient’s risk category. For reduction in CHD, EASCP considers assessing and possibly treating Lp(a) as a priority after lowering LDL-C. Studies suggest that risk of CVD-associated elevated Lp(a) levels is much higher in the presence of other CV risk factors including high LDL-C levels [29]. Several studies showed that lowering LDL-C in the presence of high Lp(a) resulted in a reduction in CVD events [30]. Thus, statins should be considered as a first-line therapy for the treatment of elevated LDL-C and Lp(a). Interestingly some studies recommend more aggressive treatment of LDL-C in the presence of high Lp(a) [2]; the effectiveness of this approach has not been fully studied. It should be noted that there is ongoing controversy about specific cholesterol targets. In patients with elevation of Lp(a) without an indication for LDL-C therapy, beneficial effect of statin for risk reduction is not clear.

Clinical studies investigating the effect of lowering Lp(a) and CHD risks seem to lack consistency in regards to patient selection, drugs used, and the method for Lp(a) measurement. LDL-C levels are frequently calculated using Friedwald equation which includes Lp(a). Using Dahlen equation, we and others have shown that in patients with high levels of Lp(a) this equation overestimates LDL-C [31, 32]. In patients with extreme Lp(a) levels this overestimation is high (up to 40 %) [32]. Thus, in a patient with very high Lp(a) level, if LDL-C is driven lower pharmacologically, a larger proportion of calculated LDL-C is actually contributed by Lp(a). The recent IMPROVE-IT study proposed that lowering LDL-C level to 1.3 mmol/L is associated with lower repeated CV events compared to the higher levels [33]. However, it is important to estimate the true levels of LDL-C in this study and its relationship with CV events.

### Treatment

Different classes of lipid-lowering medications have distinct effects on LDL and Lp(a). Statins and bile acid sequestrates reduce LDL-C, but lower Lp(a) only slightly. In patients with the familial hypercholesteremia statins decrease Lp(a) by 17–22 % [34]. Some studies showed that statins may raise Lp (a) mass by 10–50 % [6]. Fibric acid derivatives do not decrease Lp(a) except for bezafibrate which lowered Lp(a) by 39 % [35]. Bezafibrate is not approved for use in North America.

High dose of nicotinic acid (2–4 g/day) is the most effective agent that lowers Lp(a) by up to 40 % [1, 2]. Percentage lowering appears to be greater at extreme Lp(a) levels. It also has other beneficial effects including reduction of LDL-C, apoB, small LDL-C, and triglycerides, and raises HDL-C. However, no study has correlated nicotinic acid-induced reduction in Lp(a) with CVD outcomes. In Post hoc analysis of the AIM-HIGH trial, Albers et al. have suggested that despite the fact that niacin induced favorable changes in lipid profile; it did not improved CV risk [36]. They suggested that in patients with elevated Lp(a) statins remain the basis of treatment with the target of reducing LDL-C below 1.8 mmol/L. But this notion has not been studied in patients with extreme Lp(a) levels. The subsequent study, HSP-2-THRIVE study also failed to show a significant benefit in reducing major vascular events with nicotinic acid despite reduction in Lp(a) levels [37]. Also, the National Cholesterol Education Program Adult Treatment Panel III stated that the clinical utility of niacin is not fully established due to the fact that the frequency of extremely high Lp(a) level is low [20]. Interestingly, in a case report of a patient with normal lipid profile but extremely elevated Lp(a) who developed a non-ST elevation myocardial infarction, we have shown that treatment with a combination of nicotinic acid and statin may be beneficial in reducing further CHD events [38]. It is unclear if nicotinic acid has a role in reduction of CVD events. However, a recent meta-analysis suggests that nicotinic acid is useful for CV event reduction [39]. In patients with elevated Lp(a), EASCS recommends treatment with nicotinic acid 1–3 g/d in high risk patients to achieve Lp(a) levels below 500 mg/L as a secondary goal after reduction of LDL-C [12].

There are also several recent pharmacologic developments pertinent to lowering Lp(a). One is mipomersen, an apoB antisense oligonucleotide that inhibits synthesis of apoB. This drug has been approved by FDA for lowering LDL-C, apoB, TC, and non-HDL-C in patients with homozygous familial hypercholesteremia [1, 2, 40]. A meta-analysis investigating the effect of this agent on Lp(a) levels have found a reduction of 26 % in Lp(a) from baseline [1]. Due to its hepatotoxicity, this agent can only be prescribed by specially certified physicians.

Another development is human monoclonal antibody to Proprotein convertase subtilisin/kexin9 (PCSK9) [1, 2, 40]. PCSK9 is a protein which binds to LDL-receptor (LDL-R). This complex is subject to proteolytic degradation, thus preventing recycling of LDL-R to the cell surface and impairing the clearance of plasma LDL-C. The PROFICIO (Program to Reduce LDL and CV Outcomes Following Inhibition of PCSK9 In Different Populations) has shown that administration of Evolocumab, a PCSK9 inhibitor resulted in significant dose-dependent decrease in Lp(a) by up to 29.5 % and LDL-C by 52 % [41]. Alirocumab, another monoclonal antibody, showed similar results as evolocumab in terms of Lp(a) reduction [1, 40]. In a meta-analysis of 20 randomized controlled trials, Li et al. demonstrated that treatment with PCSK9 inhibitor resulted in reduction in Lp(a) as well as other lipid parameters such as LDL-C, TC, triglycerides, and apoB. Even though PCSK9 inhibitors reduce Lp(a), their effect on CVD outcome remains unclear [1, 40]. Also, it is unclear where PCSK9 inhibition fits into current therapeutic guidelines and whether this agent will be used to specifically lower Lp(a) in addition to the robust LDL-C reduction.

Lomitapide, and microsomal triglyceride transfer protein (MTP) inhibitor, approved by FDA as an adjunct to diet and lipid lowering therapy lowers LDL-C, TC, apoB, and non-HDL-C in patients with homozygous FH [42]. MTP transfers lipids to apoB in hepatocytes and enterocytes. MTP inhibition impair VLDL formation. In phase II clinical trial this agent has been shown to lower Lp(a) by 17 % [40]. There is concern about its hepatotoxicity, thus, it should be prescribed by specifically certified physicians. The long-term effect of this agent on Lp(a) reduction needs to be determined.

Inhibitors of cholesterol ester transfer protein (CETP), a plasma protein that transfers cholesterol esters from HDL to apoB-containing particles raise HDL-C levels [40]. While the first trials of these inhibitors, torcetrapib and dalcetrapib were terminated due to side effects, newer agents anacetrapib and evacetrapib showed beneficial lipid effects without serious side effects. In the DEFINE trial, anacetrapib resulted in HDL-C elevation of 138 % and lowering of LDL-C and Lp(a) by 40 and 36 %, respectively [43]. The effect of this agent on lowering CVD is under investigation.

Recently both animal and human studies have shown that specifically targeting Lp(a) with second generation antisense oligonucleotides lowers plasma level of Lp(a) by inhibiting apo(a) mRNA translation and thereby synthesis [44]. In a randomized, double-blind, placebo-controlled phase I study, Tsimikas et al. [45] have shown that treatment of volunteers with second generation antisense oligonucleotides that inhibits Lp(a) mRNA translation reduces Lp(a) levels and oxidized phospholipid associated with apo(B) levels, in a dose dependent manner, up to 89 and 93 %, respectively. They also showed this drug is safe and tolerable. It is a potent and selective in reducing Lp(a) levels, thus, it could be used as a potential therapeutic drug to reduce CVD events and progression.

Lipoprotein apheresis, an extracorporeal therapy, is the most effective way to lower Lp(a) [1, 2, 40]. Several studies have shown its lowering effect on serum lipids and Lp(a) as well as its safety [1, 40]. Currently, most lipid societies recommend apheresis mainly for patients with familial hypercholesteremia [40, 46]. The German Committee of Physicians and Health Insurance Funds and England’s HEART-UK recommend apheresis for patients with Lp(a) > 600 mg/L with progressive CHD [46, 47]. Both longitudinal cohort study and prospective observational study of patients with CHD and elevated Lp(a) levels showed that lipoprotein apheresis resulted in Lp(a) level reduction of more than 60 % after each apheresis session as well as lowering major adverse coronary events rate per patient by almost 80 % [40]. In a randomized controlled interventional study on patients receiving weekly lipoprotein apheresis, Safarova et al. demonstrated that lipoprotein apheresis resulted in Lp(a) level reduction by 73 ± 12 % [48]. Also, coronary angiography showed a reduction in median percent diameter stenosis by 2.0 compared to the group receiving only atorvastatin. Julius et al. demonstrated that lipoprotein apheresis resulted in reduction of CV events more significantly in patients with elevated Lp(a) levels compared to patients with elevated LDL-C levels [49]. Given that apheresis removes both Lp(a) and LDL-C, the therapeutic effects of apheresis seen in above studies is likely related to reduction in these particles. A major limitation of apheresis is that both Lp(a) and LDL-C levels, particularly Lp(a) levels, rebound to baseline levels within 2 weeks of treatment [40]. Other limitations include its cost and limited access.

### Future studies

Lp(a) is considered as an risk factor for CVD. In order to be used for risk stratification and as a major cardiovascular risk factor, further studies should be conducted to show Lp(a) predictive power for CVD and beneficial effects of its lowering.

## Conclusions

Epidemiologic and genetic studies provide evidence that Lp(a) is an independent, causal risk factor for CVD. Elevated Lp(a) levels promote atherosclerosis and thrombosis. Screening and treatment of selected patients are recommended. Diet, exercise, and lifestyle modification have no effect on Lp(a). Niacin and lipoprotein apheresis are the most effective ways currently available to lower Lp(a). Recent pharmaceutical developments show promising results on reduction in Lp(a) levels, but there is no study investigating their effect on CV events. Thus, large prospective randomized-controlled trials are needed to investigate relationship between lowering Lp(a) and reduction in CVD.
